# Supplementary material for: Burnout experience among healthcare workers post third COVID-19 wave in India; findings of a cross-sectional study
Source: PeerJ. 2024 Sep 9;12:e18039. doi: 10.7717/peerj.18039 (PMC11391938; doi:10.7717/peerj.18039)
Supplement: Supplemental Information 3 [file peerj-12-18039-s003.docx]

**Questionnaire;** Stress and burnout experience among healthcare workers post third COVID-19 wave in India; findings of a cross-sectional study

*Please confirm if you have filled the consent form and approved your participation

Yes O No O

*Please confirm, if you were involved in direct patient care during the 3^rd^ wave of COVID-19 in India.

Yes O No O

*General instructions: Please tick most suitable option provided and tick only one option always. We assure confidentiality of your responses. Hence, we kindly request you to respond promptly. Thank you*

| **Survey informations** | | | |
| --- | --- | --- | --- |
| 1 | Date of filling the survey form |  |  |
| 2 | On average how much time it took to fill this form? |  |  |
| 3 | Please mention the district of your clinical practice and State? |  |  |
| 4 | Do you suffer from any physical ailments? | Yes O No O |  |

| 5 | What is age? | In years |  |
| --- | --- | --- | --- |
| 6 | Please specify your sex? | Male O Female O  Prefer not to say O |  |
| 7 | Please choose your appropriate marital status? | Not married yet O  Married O  Divorced/singled O |  |
| 8 | Do you have children? If yes specify the number please? | Yes O No O  If yes, ______number |  |
| 9 | Please select your profession from the list | Surgeon O  Physician O  Medical resident O  Dentists O  Dentistry resident O  Nurse O  Physiotherapist O  Technician O  Others: please specify  _------------------_--------- |  |
| 10 | Please specify your overall work experience? | _____________ years |  |
| 11 | What are your working hours during the 3^rd^ wave of COVID19 in a typical week? | ____________hours/week |  |
| 12 | Did you work as a frontline worker during the 3^rd^ wave of COVID19? | Yes O No O |  |
| 13 | Which of the following work shifts best applies to your work shift during the 3^rd^ wave of COVID19? | Day shift O  Night shift O  Both/Alternative O |  |
| 14 | Were you test positive for COVID-19 in the now/past? | Yes O No O |  |
| 15 | Please specify, your BMI category based on your recent measurements? | Underweight O  Normal O  Overweight O  Obese O |  |

| **The Copenhagen Burnout Inventory; Part 1 Personal burnout**  “feelings of exhaustion and fatigue on a personal level” | | | | | |
| --- | --- | --- | --- | --- | --- |
| *Please tick one option only* | Always | Often | Sometimes | Seldom | Never/almost never |
| How often do you feel tired | O | O | O | O | O |
| How often are you physically exhausted? | O | O | O | O | O |
| How often are you emotionally exhausted? | O | O | O | O | O |
| How often do you think: “I can take it anymore”? | O | O | O | O | O |
| How often do you feel worn out? | O | O | O | O | O |
| How often do you feel weak and susceptible to illness? | O | O | O | O | O |
| **The Copenhagen Burnout Inventory; Part 2 Work burnout**  Work burnout is a state of prolonged physical and Psychological exhaustion, which is perceived as related to the person's work. | | | | | |
|  | Always | Often | Sometimes | Seldom | Never/almost never |
| Is your work emotionally exhausting? | O | O | O | O | O |
| Do you feel burnt out because of your work? | O | O | O | O | O |
| Does your work frustrate you? | O | O | O | O | O |
| Do you feel worn out at the end of the working day? | O | O | O | O | O |
| Are you exhausted in the morning at the thought of another day at work? | O | O | O | O | O |
| Do you feel that every working hour is tiring for you? | O | O | O | O | O |
| Do you have enough energy for family & friends during leisure time? | O | O | O | O | O |
| **The Copenhagen Burnout Inventory; Part 3 Client burnout**  state of Prolonged physical and psychological exhaustion, which is  perceived as related to the person's work with clients | | | | | |
|  | To very high degree | To a high degree | Somewhat | To a low degree | To a very low degree |
| Do you find it hard to work with client | O | O | O | O | O |
| Do you feel it frustrating to work with client? | O | O | O | O | O |
| Does it drain your energy to work with client? | O | O | O | O | O |
| Do you feel that you give more than you get back when you work with clients? | O | O | O | O | O |
| Are you tired of working with clients? | O | O | O | O | O |
| Do you sometimes wonder how long you will be able to continue working with clients? | O | O | O | O | O |

Thank you for your time and responses
