# Supplementary material for: Burnout experience among healthcare workers post third COVID-19 wave in India; findings of a cross-sectional study
Source: PeerJ. 2024 Sep 9;12:e18039. doi: 10.7717/peerj.18039 (PMC11391938; doi:10.7717/peerj.18039)
Supplement: Supplemental Information 4 [file peerj-12-18039-s004.pdf]

**Questionnaire;** Stress and burnout experience among healthcare workers post third COVID-19 wave in India; findings of a cross-sectional study

*Please confirm if you have filled the consent form and approved your participation

Yes O        No O

*Please confirm, if you were involved in direct patient care during the 3rd wave of COVID-19 in India.

Yes O        No O

*General instructions: Please tick most suitable option provided and tick only one option always. We assure confidentiality of your responses. Hence, we kindly request you to respond promptly. Thank you*

| **Survey informations** | | | |
|---|---|---|---|
| 1 | Date of filling the survey form | | |
| 2 | On average how much time it took to fill this form? | | |
| 3 | Please mention the district of your clinical practice and State? | | |
| 4 | Do you suffer from any physical ailments? | Yes O     No O | |

| | | | |
|---|---|---|---|
| 5 | What is age? | In years | |
| 6 | Please specify your sex? | Male O  Female O<br>Prefer not to say O | |
| 7 | Please choose your appropriate marital status? | Not married yet O<br>Married O<br>Divorced/singled O | |
| 8 | Do you have children? If yes specify the number please? | Yes O     No O<br>If yes, ______number | |
| 9 | Please select your profession from the list | Surgeon O<br>Physician O<br>Medical resident O<br>Dentists O<br>Dentistry resident O<br>Nurse O<br>Physiotherapist O | |

| | | | |
|---|---|---|---|
| | | Technician O<br>Others: please specify<br>________________ ________ | |
| 10 | Please specify your overall work experience? | ______________ years | |
| 11 | What are your working hours during the 3rd wave of COVID19 in a typical week? | ____________hours/week | |
| 12 | Did you work as a frontline worker during the 3rd wave of COVID19? | Yes O No O | |
| 13 | Which of the following work shifts best applies to your work shift during the 3rd wave of COVID19? | Day shift O<br>Night shift O<br>Both/Alternative O | |
| 14 | Were you test positive for COVID-19 in the now/past? | Yes O No O | |
| 15 | Please specify, your BMI category based on your recent measurements? | Underweight O<br>Normal O<br>Overweight O<br>Obese O | |

**The Copenhagen Burnout Inventory; Part 1 Personal burnout**

"feelings of exhaustion and fatigue on a personal level"

| *Please tick one option only* | Always | Often | Sometimes | Seldom | Never/almost never |
|---|---|---|---|---|---|
| *How often do you feel tired* | O | O | O | O | O |
| *How often are you physically exhausted?* | O | O | O | O | O |
| *How often are you emotionally exhausted?* | O | O | O | O | O |
| *How often do you think: "I can take it anymore"?* | O | O | O | O | O |
| *How often do you feel worn out?* | O | O | O | O | O |
| *How often do you feel weak and susceptible to illness?* | O | O | O | O | O |

**The Copenhagen Burnout Inventory; Part 2 Work burnout**

Work burnout is a state of prolonged physical and Psychological exhaustion, which is perceived as related to the person's work.

| | Always | Often | Sometimes | Seldom | Never/almost never |
|---|---|---|---|---|---|
| *Is your work emotionally exhausting?* | O | O | O | O | O |

| | | | | | |
|---|---|---|---|---|---|
| *Do you feel burnt out because of your work?* | O | O | O | O | O |
| *Does your work frustrate you?* | O | O | O | O | O |
| *Do you feel worn out at the end of the working day?* | O | O | O | O | O |
| *Are you exhausted in the morning at the thought of another day at work?* | O | O | O | O | O |
| *Do you feel that every working hour is tiring for you?* | O | O | O | O | O |
| *Do you have enough energy for family & friends during leisure time?* | O | O | O | O | O |

**The Copenhagen Burnout Inventory; Part 3 Client burnout**

state of Prolonged physical and psychological exhaustion, which is perceived as related to the person's work with clients

| | To very high degree | To a high degree | Somewhat | To a low degree | To a very low degree |
|---|---|---|---|---|---|
| *Do you find it hard to work with client* | O | O | O | O | O |
| *Do you feel it frustrating to work with client?* | O | O | O | O | O |
| *Does it drain your energy to work with client?* | O | O | O | O | O |
| *Do you feel that you give more than you get back when you work with clients?* | O | O | O | O | O |
| *Are you tired of working with clients?* | O | O | O | O | O |
| *Do you sometimes wonder how long you will be able to continue working with clients?* | O | O | O | O | O |

Thank you for your time and responses
