# Supplementary material for: Burnout experience among healthcare workers post third COVID-19 wave in India; findings of a cross-sectional study
Source: PeerJ. 2024 Sep 9;12:e18039. doi: 10.7717/peerj.18039 (PMC11391938; doi:10.7717/peerj.18039)
Supplement: Supplemental Information 5 [file peerj-12-18039-s005.docx]

Title: **Stress and burnout experience among healthcare workers post third COVID-19 wave in India; findings of a cross-sectional study**

**STROBE** Statement—checklist of items that should be included in reports of ***cross-sectional studies***

|  | Item No | Recommendation | Page |
| --- | --- | --- | --- |
| **Title and abstract** | 1 | 1. Study design is indicated in the title, abstract, methods section as *web-based cross-sectional study design.* | 1 |
|  |  | (b) A balanced summary of what was done and what was found is provided in the methods and result section of the abstract respectively. | 1 |
| Introduction | | |  |
| Background/rationale | 2 | The scientific background and rationale for the investigation is reported in para 2, 3, and 4. | 2-3 |
| Objectives | 3 | Specific objectives are stated as the final sentence of last (4^th^) paragraph under background section. | 3 |
| Methods | | |  |
| Study design | 4 | Mentioned in the first sentence of the first para of method section under *sub-section study design .* | 3 |
| Setting | 5 | A detailed description of the study area (Jaipur district), and population healthcare professionals), study period, and the description of study population is mentioned under sub-section study area under Method section *(2 para of methods).* | 4 |
| Participants | 6 | Eligibility criteria (sub-sec5tion; sample size & eligibility criteria), the sources and methods of selection of participants are clearly stated in the 2^nd^ paragraph of methods section. | 5 |
| Variables | 7 | The outcome variable (CBI score and the predictor variables of burnout are reported in the 4th para of methods section. | 4 |
| Data sources/ measurement | 8 | Source of data and data analysis methods are described in the under method section, in the data analysis sub-section. | 5 |
| Bias | 9 | Efforts to address potential sources of bias were described in several part of method session. | 1-5 |
| Study size | 10 | Study size determination and the flow of sampling procedure is mentioned 4^th^ para of method section. | 5 |
| Quantitative variables | 11 | All quantitative variables treated as qualitative after categorizing them in one of most commonly used categories. | 5 |
| Statistical methods | 12 | (*a*) Statistical methods used in this study are described under data analysis sub-section in the last para of method session. | 5 |
|  |  | (*b*) Both sub group analysis and interaction terms were used. | 5 |
|  |  | (*c*) There were no missing data in this study | 5 |
|  |  | *(d)* Not applicable |  |
|  |  | (*e*) Not applicable |  |
| Results | | |  |
| Participants | 13 | (a) Number of participants, response rate is presented in the first paragraph of results session and detailed socio-demographic characteristics and frequency distribution in the table 1. | 6 |
|  |  | (b) About 50.17% participants responded, and mentioned in the first para of result section. | 6 |
|  |  | (c) This was cross-sectional study so; there is no flow as that of longitudinal study. | 6 |
| Descriptive data | 14 | (a) Characteristics of study participants (eg demographic, clinical, social) is presented in tables 1 | 6 |
|  |  | (b) There were no missing data in this study | 6 |
| Outcome data | 15 | Outcome variable (Copenhagen burnout inventory (CBI)) described and summarized in methods section. | 5-6 |
| Main results | 16 | (*a*) Unadjusted estimates and confounder-adjusted estimates and their precision (eg, 95% confidence interval) are presented in table 3 and 4. Discussed *Factors associated with burnout* 1^st^ para in result section. | 6-10 |
|  |  | 1. Category boundaries of continuous variables were categorized and reported in all tables. |  |
|  |  | (*c*) Regression model was used and expressed in odds ratio. | 8-10 |
| Other analyses | 17 | No clear or significant sub group difference noted and interaction terms were used but non-significant. | 6 |
| Discussion | | |  |
| Key results | 18 | Key results to study objectives are discussed under discussion session with references. | 11 |
| Limitations | 19 | Limitations and possible strengths related to the current study are discussed in the final paragraph of discussion session on the way of viewing direction for researchers. | 13 |
| Interpretation | 20 | A cautious overall interpretation of results considering objectives, results from similar studies, and other relevant evidence is discussed under limitation para of discussion session. | 13 |
| Generalisability | 21 | Generalisability (external validity) of the study results are mentioned under conclusion section | 15 |
| Other information | | |  |
| Funding | 22 | Information regarding the source of funding is reported in the declaration section. |  |
