# Supplementary material for: Burnout experience among healthcare workers post third COVID-19 wave in India; findings of a cross-sectional study
Source: PeerJ. 2024 Sep 9;12:e18039. doi: 10.7717/peerj.18039 (PMC11391938; doi:10.7717/peerj.18039)

**Figure**

**Distribution of burnout according to health profession**

1. **Personal**


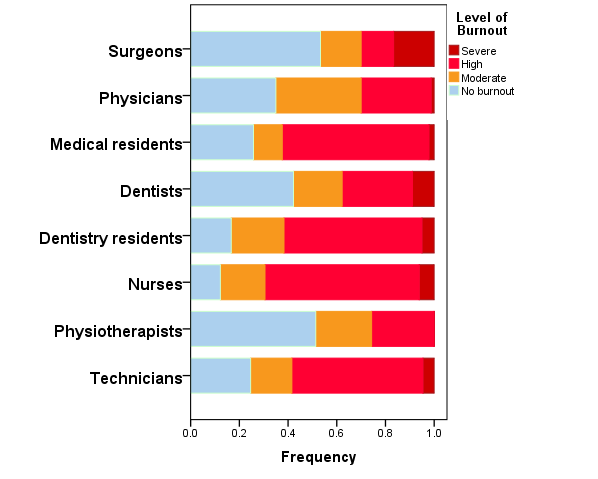


1. **Work related**


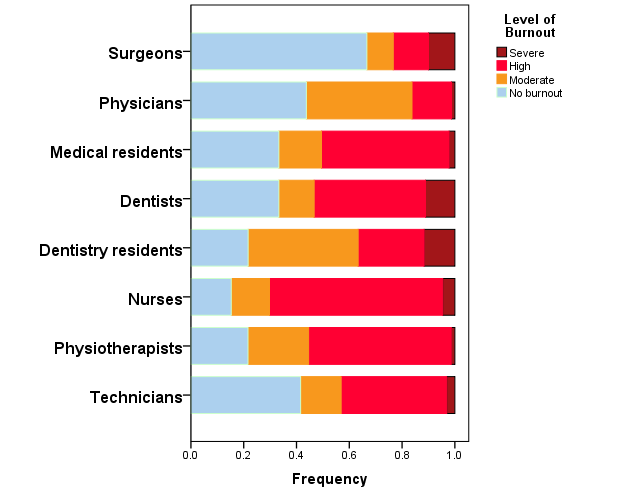


1. **Client related**


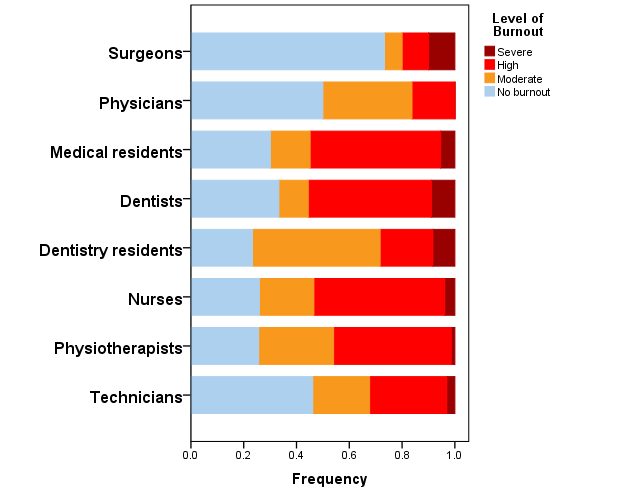

Supplement: Supplemental Information 6 [file peerj-12-18039-s006.docx]
